# Multiscale Static Compressive Damage Characteristics of Kiwifruit Based on the Finite Element Method

**DOI:** 10.3390/foods13050785

**Published:** 2024-03-03

**Authors:** Yue Zhu, Licheng Zhu, Wangkun Guo, Zhenhao Han, Ruixue Wang, Weipeng Zhang, Yanwei Yuan, Jianbo Gao, Suchun Liu

**Affiliations:** 1 National Key Laboratory of Agricultural Equipment Technology, Chinese Academy of Agricultural Mechanization Sciences Group Co., Ltd., Beijing 100083, China; zhuyue920@gmail.com (Y.Z.); zhulicheng@caams.org.cn (L.Z.); hanzhenhao@caams.org.cn (Z.H.); wangruixue@caams.org.cn (R.W.); zhwellpeng@gmail.com (W.Z.); jianbogao121@163.com (J.G.); suchun1804@163.com (S.L.); 2 College of Astronautics, Northwestern Polytechnic University, Xi’an 710129, China; gwangkun@163.com

**Keywords:** kiwifruit, mechanical properties, multiscale, compression, damage finite element simulation

## Abstract

In the handling or processing process, fruits are easily crushed by external loads. This type of damage in fruit often leads to the internal pulp browning and rotting, with the severity largely dependent on the fruit tissue’s geometric and mechanical properties. In kiwifruits, with their thin skin and dark-colored flesh, it is particularly challenging to observe and analyze the damage caused by extrusion through traditional experimental methods. The objective of this research is to construct a multi-scale finite element model encompassing the skin, flesh, and core by measuring the geometric and mechanical properties of kiwifruit, to assess and predict the damage characteristics under compression, and to verify the accuracy of the finite element model through experiments. The results indicated that kiwifruits demonstrated different compressive strengths in different directions during compression. The compressive strength in the axial direction was higher than that in the radial direction, and there was little difference between the long and short radial directions. The flesh tissue is the most vulnerable to mechanical damage under external compression, followed by the core. At strain levels below 5%, there was no noticeable damage in the axial or radial directions of the kiwifruit. However, when strain exceeded 5%, damage began to manifest in some of the flesh tissue. To maintain fruit quality during storage and transportation, the stacking height should not exceed 77 fruits in the axial direction, 48 in the long direction, and 53 in the short direction. The finite element analysis showed that the established model can effectively simulate and predict the internal damage behavior of kiwifruits under compression loads, which is helpful for a deeper understanding of the mechanical properties of fruits and provides a theoretical basis and technical guidance for minimizing mechanical damage during fruit handling.

## 1. Introduction

Kiwifruit is a nutritionally rich fruit that is widely cultivated around the world [1]. According to data from the Food and Agriculture Organization of the United Nations (FAO), in 2021, the global planting area of kiwifruit was 2.86 million hectares, with a production of 4.47 million tons, mainly in China, New Zealand, Italy, and Chile [2]. It contains rich components such as vitamin C, vitamin E, dietary fiber, etc., with anti-aging and enhanced immunity effects [3]. Fruits are prone to compressive and impact loads during harvesting, grading, packaging, transportation, and storage. When the stress exceeds the bioyield stress of the fruit tissue, it will cause mechanical damage to different degrees, which will lead to changes in the shape, color, flavor, and nutritional components of the fruit and affect its economic value [4,5]. Meanwhile, the damage characteristics of the fruits are also influenced by factors such as the maturity and storage conditions [6]. Therefore, compressive damage caused by external forces during the handling process has become a major problem to be solved. It was found that compressive damage is an important factor in the softening and rotting of fruits after harvest, and static compressive damage is one of the most common forms of damage [7]. When compressive loads act on kiwifruit, the fruit may show a certain degree of elastic deformation at the beginning of compression. With the increase in pressure, the deformation exceeds the elastic limit of the pulp cells, and the fruit begins to exhibit plastic deformation, and the interior of the fruit begins to be damaged, accompanied by the rupture of the skin and the outflow of juice. Therefore, the reduction in mechanical damage to kiwifruit during picking, transportation, and storage has become an important research topic in order to thoroughly understand its mechanical properties and the damage mechanism under loads.

Kiwifruit is a post-ripening fruit which is hard at harvest and can withstand a certain amount of compressive force without damage. The shape of kiwifruit is irregular, mainly consisting of the skin, flesh, and core. The tissue material exhibits anisotropic characteristics, and its micro-scale structure is a composite material, with mechanical properties varying in different directions. Understanding the effects of these differences on the mechanical properties of kiwifruit is beneficial for reducing compressive damage. In traditional fruit damage research, the damage behavior of fruits is mainly predicted and simulated through drop tests [8], impact experiments [9], compression tests [10], and pendulum experiments [11]. However, these experimental methods are complex, time-consuming, and costly. For fruits with irregular geometric shapes, it is difficult to accurately describe internal damage under external loads through these methods. In recent years, hyperspectral imaging technology has also been used to detect mechanical damage in fruit. Hyperspectral detection technology is mainly used for non-destructively assessing maturity, quality, and internal damage by analyzing the spectral information of reflection or transmission at different wavelengths, and is suitable for rapid and large-scale fruit quality inspection and grading [12,13]. However, this method has high requirements for fruit appearance, optical properties, and damage types. Qiang Lü et al. [14] used hyperspectral imaging technology to detect latent damage in kiwifruit and proposed a method for the early detection of latent damage based on hyperspectral imaging technology. Experimental results showed an error of 14.5% in detecting latent damage in fruits. However, based on limited sample data, this might affect the generalization ability of the model, and the high costs and operational complexity may limit its popularization in small-scale agricultural production. Hongyan Zhu et al. [15] investigated the feasibility and potential of measuring the hardness, soluble solid content, and pH value of kiwifruit through variable selection methods and calibration models, and predicted the internal quality of kiwifruit based on hyperspectral imaging technology. In the study, complex chemometric models and variable selection algorithms were used which challenged the quality assessment of kiwifruit under specific storage conditions. Jing Li et al. [16] explored the method of non-destructive detection of kiwifruit texture features by using near-infrared hyperspectral imaging technology, and emphasized the role of hyperspectral imaging technology in improving the efficiency of agricultural product classification and quality assessment. However, there are certain limitations in real-time detection capability and cost-effectiveness analysis, including difficulties in the application to different types of fruits or production environments, and no discussion on cost–benefit factor. Yuchen Zhao et al. [17] used deep learning and hyperspectral imaging technology for quality classification research on kiwifruit under different storage conditions. The results showed that hyperspectral imaging combined with artificial intelligence technology can effectively assess the quality changes of kiwifruit under different storage conditions. In the study, deep learning models were used to improve prediction accuracy, but the training of models requires high computational costs and has shortcomings in different practical application scenarios.

The Finite Element Method (FEM) is an efficient numerical calculation method that can handle complex boundary conditions and nonlinear problems well. It is widely used to predict the damage behavior of agricultural products under external forces [18]. Kunpeng Tian et al. [19] studied the mechanical properties of SunGold kiwifruit and simulated the damage behavior of SunGold kiwifruit under compression by the finite element method. The results showed that the model can simulate the stress distribution, deformation, and damage range of the fruit under different compression forces, providing an effective method for predicting the harvest damage of kiwifruit. In the study, the fruit is regarded as an object with a regular shape for simulation, which may affect the accuracy of the model, especially in the prediction of stress distribution. In reality, kiwifruit often has an irregular shape and complex internal structure which significantly affect the stress response and damage distribution of the fruit when subjected to external forces. Zhiguo Li et al. [20] used a multi-scale finite element model to predict the mechanical damage of various tissues of tomatoes under external pressure. The results showed that this method is highly accurate and can be applied to the internal mechanical damage research of other fruits and vegetables. Wei Liu et al. [21] established a finite element model of internal mechanical damage during pineapple compression based on Hooke’s Law and Hertz’s Law. The results showed that the established finite element model can truly reflect the compression damage behavior of pineapples. Jian Zhao et al. [22] used FEM explicit dynamics to simulate and predict the collision bruising of fresh goji berries. The results showed that when the drop height was 0.2~0.5 m, the impact angle was 10~30°, and the impact material was foam board, wood board, or nylon board, there was no damage to the goji berries. Alireza Salarikia et al. [23] used FEM to establish a finite element simulation model of pear dropping. The results showed that the finite element simulation stress and strain were consistent with the experimental values. Yancheng Lang et al. [24] used the finite element method to simulate the mechanical damage of prickly pear under compressive load. The numerical results of the finite element simulation were in good agreement with the experimental results. In the above research, most fruit damage models were based on linear elasticity, treating fruit tissue as an isotropic material, and ignoring the impact of the core and skin on the damage characteristics of the fruit. In fact, most fruit tissues have anisotropic characteristics, and the damage characteristics of the fruit are closely related to its tissue material and geometric shape [25]. To obtain accurate simulation results, it is necessary to measure the mechanical properties of the fruit and establish a multi-scale finite element model.

In this research, the geometric and mechanical properties and compression of kiwifruit were studied by combining multi-scale finite element modeling with experimental techniques. Initially, the mechanical parameters of various tissues of kiwifruit (including the skin, flesh, and core) were determined through mechanical experiments. Then, with the help of advanced 3D reverse engineering technology, a three-dimensional geometric model of kiwifruit containing the skin, flesh, and core was accurately constructed. Next, Abaqus finite element simulation software was used to solve the model, and the stress distribution, deformation, and damage of the fruit under compressive loads were obtained, and the simulation results were compared and analyzed with experimental data. The purpose of the study is to accurately obtain the mechanical parameters of kiwifruit tissue materials through mechanical experiments and to discuss the stress distribution under compressive loads. The core objective is to determine the compressive damage threshold of the fruit, and this has significant theoretical and technical implications for reducing compressive damage to kiwifruit, prolonging the shelf life, and optimizing the structure of harvesting equipment. Specifically, the goals of this study include:

(1) Determining the mechanical parameters of kiwifruit’s skin, flesh, and core through experiments.
(2) Establishing a multi-scale kiwifruit compression finite element model that includes the skin, flesh, and core.
(3) Simulating the stress–strain distribution and damage characteristics of kiwifruit under static compressive loads using finite element simulation software.
(4) Validating the accuracy of the model by comparing simulation results with experimental data.
(5) Determining the damage threshold of kiwifruit, providing theoretical and technical support for the reduction in compression damage.

## 2. Materials and Methods

### 2.1. Materials and Equipment

**Figure 1:**
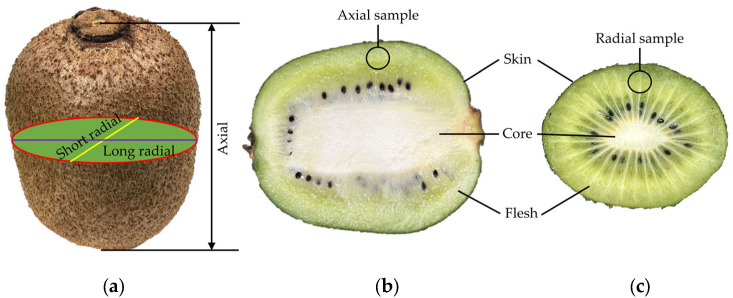
Kiwifruit structure. (**a**) External morphology; (**b**) axial sample; (**c**) radial sample.

In this paper, fresh and ripe “Xuxiang” kiwifruit was selected as the research object, and was collected from the orchard in Zhouzhi County Shanxi Province (longitude: 108°22′ E, latitude: 344°18″ E). All fruits were manually harvested to ensure that the flesh tissue was free of any damage. The harvested kiwifruits were stored in an incubator at a temperature of 25 °C and a relative humidity of 80% for later use. An electronic universal testing machine (DF22-102T, China Central Machinery Test Equipment Co., Ltd., Beijing, China) equipped with a 250 N pressure sensor, with a measurement error of ±0.5%, was used in the experiments, and the experimental data were automatically recorded by a computer. The instruments required for the experiment also included a vernier caliper (accuracy 0.01 mm), electronic balance (accuracy 0.01 g), blades, and a homemade flesh sampler. Before the experiment, 80 fruits of similar appearance, close mass, and without any damage were randomly selected, labeled, and placed in an indoor environment for 2 h. The basic dimensions of the fruits in the axial, long radial, and short radial directions were measured separately with a vernier caliper, as shown in Figure 1a. Usually, the fibers are reinforced in the axial direction of kiwifruit, resulting in greater mechanical properties axially than radially. Therefore, it is necessary to measure the mechanical properties of the flesh in both axial and radial directions separately. The fruit was cut along the axial and radial directions. The internal structure of the fruit and the location of the flesh samples are shown in Figure 1b,c.

### 2.2. Measurement of Geometrical and Mechanical Properties of Fruit

The structure of kiwifruit mainly includes three parts: skin, flesh, and core. The flesh accounts for about 90% of the total volume of the fruit, while the skin is relatively thin but possesses good elasticity and toughness. Serving as a protective layer for the flesh, the skin plays a crucial role in preventing damage to the flesh. Embedded within the fruit, the core is primarily composed of fibrous structures. Typically, the core is harder than the flesh, providing support to the fruit’s structure and preventing deformation. In this paper, the mechanical parameters of the skin, flesh, and core were measured through tensile and compression tests. Kiwifruit skin and core have a relatively uniform tissue structure and are considered isotropic. However, the flesh, composed of various types of cells, cellulose, and pectin, exhibits anisotropic mechanical properties. Therefore, it is necessary to conduct axial and radial mechanical experiments on the flesh to fully understand its mechanical properties in different directions.

**Figure 2:**
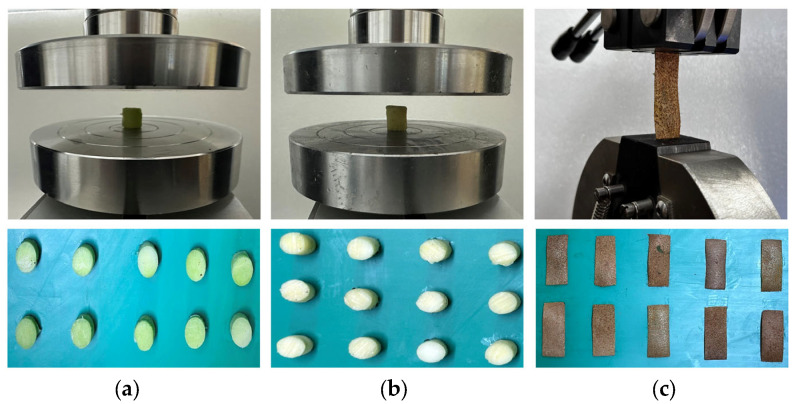
Mechanical properties test. (**a**) Pulp compression; (**b**) core compression; (**c**) skin stretching.

In this study, 20 fruits were randomly selected from the samples. The skin was cut axially into 10 rectangular samples, with lengths of 50 ± 0.50 mm, widths of 15 ± 0.50 mm, and thicknesses of 0.5 ± 0.05 mm. The flesh was cut axially and radially into 10 cylindrical samples, each with heights and diameters of 12 ± 0.50 mm and 10 ± 0.50 mm, respectively. Using the same method, the remaining cores were cut into 10 cylindrical samples of the same dimensions. For the compression test, the flesh and core samples were placed in the center of the compression plate, and the universal testing machine was set with parameters to compress the samples at a constant loading rate of 3 mm/min, ceasing the load when the sample fails under compression. Because the skin sample is thin, it is difficult to conduct compression experiments. Therefore, tensile tests were used to obtain the mechanical parameters of the skin. For the tensile test, the skin samples were fixed between the upper and lower fixtures of the universal testing machine. The machine was set with parameters to stretch the skin samples at a loading rate of 3 mm/min. The skin samples that broke near the middle area were selected as valid tensile specimens. Each experiment was repeated 10 times, and the average values were recorded as the experimental results. The experimental setup and samples are shown in Figure 2. According to Equations (1)–(3), the stress, strain, and modulus of elasticity values for the skin, flesh, and core were calculated.

(1) $$\sigma =\frac{F}{S}$$

(2) $$\varepsilon =\frac{∆L}{L}$$

(3) $$E=\frac{\sigma }{\varepsilon }=\frac{FL}{S∆L}$$

where σ is stress (MPa); ε is the tensile (compressive) strain (%); E is elastic modulus (MPa); F is the test load (N); L is the initial length (mm); S is the cross-sectional area of the material in the direction of tension (compression) (mm^2^); and ∆L is the elongation length (mm).

### 2.3. Kiwifruit Compression Experiment

**Figure 3:**
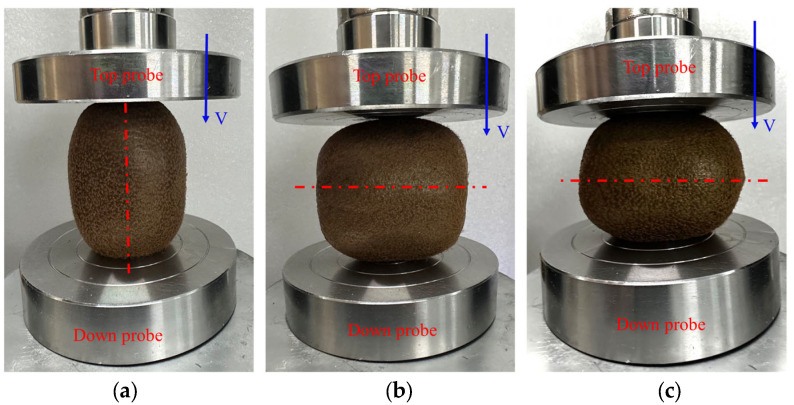
Whole kiwifruit compression test. (**a**) Axial; (**b**) long radial; (**c**) short radial.

During the harvesting and transportation of kiwifruit, it is mainly subjected to compression from external loads in the axial and radial directions. Therefore, compression experiments were conducted in three directions: axial, long radial, and short radial. In the experiment of different strain levels, kiwifruit was placed in the middle of two parallel compression plates, and four strain levels of 2.5%, 5%, 10%, and 20% were applied along the axial, long radial, and short radial directions, respectively. The working parameters of the electronic universal testing machine were set to compress the fruit at a loading rate of 3 mm/min and stopped when it reached the strain level. To verify the validity of the finite element model, the whole fruit compression yielding experiments were conducted according to the aforementioned methods. In the whole fruit compression test, a loading rate of 3 mm/min was applied in the axial, long radial, and short radial directions, stopping when the fruit yielded to compression. The experimental data were automatically recorded by the universal testing machine and collected and stored by a connected computer. To ensure the representativeness and reliability of the data, five kiwifruits of similar geometric shapes were selected for compression testing in each experiment. The direction of fruit compression and specific experimental setup are shown in Figure 3.

### 2.4. Finite Element Modeling and Simulation

#### 2.4.1. Geometric Model Construction

To enhance the feasibility of the research and reduce the complexity of the model, based on the characteristics of the kiwifruit’s tissue structure, the impact of seeds on the overall structure of the fruit was ignored in the study and it was assumed that there was a perfect adhesion between the core and the flesh without any separation. Moreover, a multi-scale three-dimensional geometric model of the kiwifruit including the skin, flesh, and core was established by embedding the core into the flesh as an independent geometric entity and setting the skin as a uniform thin shell element with a thickness of 0.5 mm. Accurate geometric entity modeling is the key to improving the predictive accuracy of the finite element model. In view of the irregular shape of the kiwifruit, a 3D laser scanner (Solutionix Rexcan III, Seongbuk-gu, Seoul, South Korea) was used along with reverse engineering technology to precisely model the shape of the fruit. A fruit was randomly selected from the experimental samples to collect surface point cloud data. The open-source software MeshLab 2021.07 (Visual Computing Lab, Pisa, Italy) was used to reconstruct the point cloud data of the fruit. Then, the processed point cloud mesh surface model was imported into Geomagic Studio (Version 2017, Geomagic, Inc., Morrisville, NC, USA) for further processing to refine the details of the fruit surface. Finally, the geometric entity modeling of the kiwifruit and its core was completed using SOLIDWORKS 2021 (Dassault Systemes S. A, Waltham, MA, USA) 3D modeling software.

#### 2.4.2. Finite Element Modeling and Meshing

**Figure 4:**
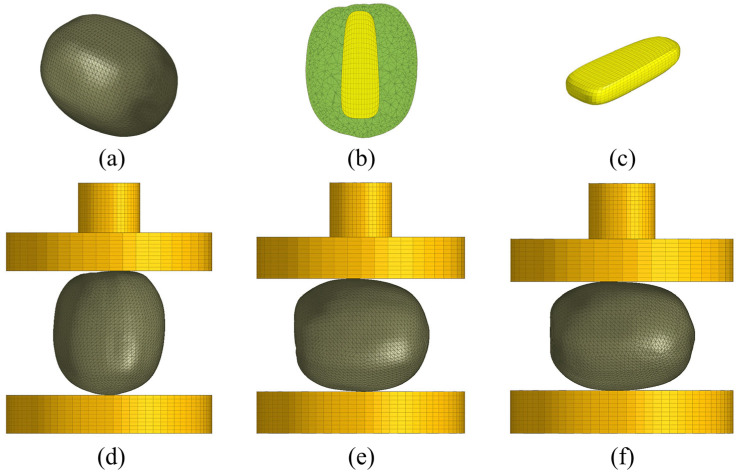
The finite element model mesh details and compression test simulation setup. (**a**) The kiwifruit finite element model; (**b**) cross-section diagram; (**c**) core; (**d**) axial compression; (**e**) long radial compression; and (**f**) short radial compression.

With the development of computer modeling technology, the finite element analysis method has been widely used in the prediction of fruit and vegetable mechanical damage, which can simulate complex structures and working conditions [26]. The basic principle of the finite element method is to divide the complex physical system into countless small units, and then calculate each unit, and finally combine the results of each unit calculation to obtain the calculation results of the entire system [27]. In this study, the commercial software Abaqus 2021/CAE (Dassault Systémes, Vélicy-Villacoublay, France) was used for finite element modeling and simulation analysis. In view of the uniform tissue structure of kiwifruit skin and core, it was set as isotropic material, while the pulp was regarded as an orthotropic material. The geometric structure of kiwifruit is irregular. The skin and pulp were meshed with the tetrahedral structural unit (C3D4), and the core part was the hexahedral structural unit (C3D8R). These two types of mesh elements can adapt to different material characteristics and complex load conditions and can provide accurate mechanical behavior simulation for irregular geometric models. In the simulation calculation, the finer grid element is beneficial to improve the calculation accuracy, but will also increase the solution time of the model. Therefore, it is very important to select the appropriate mesh size for accurate and reliable finite element numerical simulation. In this study, Hypermesh software in Altair’s HyperWorks 2021 was used to preprocess the grid, and the grid of kiwifruit skin, pulp, and core was processed independently to ensure the grid size and quality. The finite element model has 43,227 nodes and 91,380 elements. The mesh details and compression direction are shown in Figure 4.

#### 2.4.3. Boundary Conditions and Solutions

Two parallel rigid plates were used to simulate the compression damage of fruits. The rigid target unit was used to establish the contact pair between the fruit and the pressure plate, and the contact type between the fruit and the pressure plate was set as friction contact, with a friction coefficient of 0.428 [28]. The lower platen was set as a fixed constraint and did not rotate or move in all directions. Four strain levels of 2.5%, 5%, 10%, and 20% were applied to the upper platen along the axial direction, long radial, and short radial direction of the kiwifruit. During the loading process, the damage behavior of the fruit tissue can be predicted using the Von Mises yield criterion. This method is widely applied in the field of materials mechanics, and is mainly used to assess and predict the internal stress state of materials under load to evaluate the yield behavior and damage conditions of the materials [29]. In this study, based on this criterion, the damage and yield behavior of kiwifruit under compression load were analyzed and predicted. The finite element model was solved by using the explicit dynamics method in Abaqus software. The compression force–displacement curve for the kiwifruit was obtained after processing the simulation results. The simulated results were compared and analyzed with experimental data to assess the accuracy of the established model.

## 3. Results and Discussion

### 3.1. Geometrical and Mechanical Properties of Fruits

**Figure 5:**
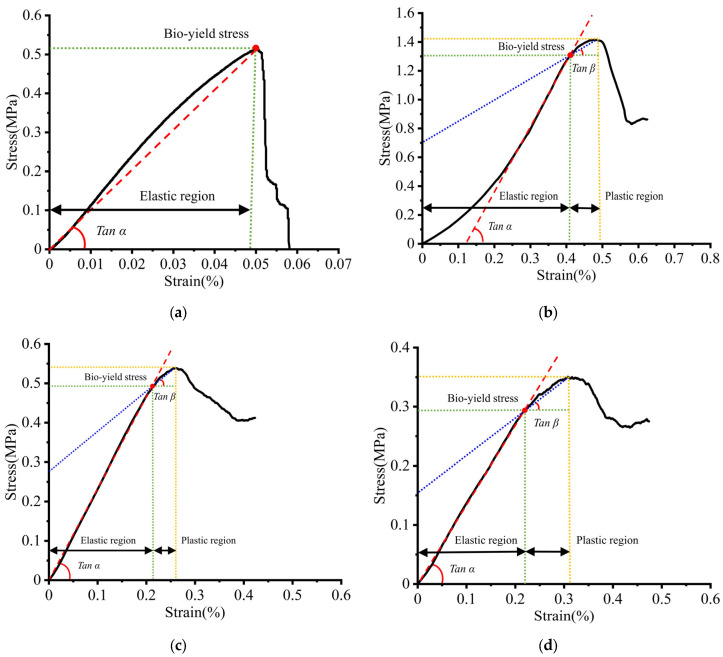
Mechanical property test. (**a**) tensile stress-strain curve of skin; (**b**) compressive stress-strain curve of core; (**c**) flesh axial compression stress-strain curve; (**d**) flesh radial compression stress-strain curve.

Through the measurement of kiwifruit’s geometric parameters, the average lengths of the fruit’s axial, long radial, and short radial directions were found to be 65.83 ± 3.62 mm, 55.43 ± 2.56 mm, and 51.16 ± 2.27 mm, respectively. The average lengths of the core’s axial, long radial and short radial directions were 52.42 ± 2.13 mm, 18.41 ± 1.86 mm, and 9.79 ± 1.02 mm, respectively, with the fruit’s mass averaging at 97.11 ± 4.83 g. The average densities of the skin, flesh, and core were 0.99 ± 0.03 g/mm^3^, 1.24 ± 0.083 g/mm^3^, and 1.15 ± 0.08 g/mm^3^, respectively. Mechanical tests provided the stress–strain relationships for the kiwifruit’s skin, core, and flesh tissues, as illustrated in Figure 5. The skin exhibited a clear elastic deformation phase during stretching. From Figure 5a, it can be seen that the stress–strain curve of the skin in the elastic region is linear, without a distinct biological yield point, showing characteristics of brittle materials. The skin ruptures directly after stretching to a certain extent, with stress subsequently decreasing.

In the compression process, the core and flesh showed clear biological yield points. The flesh displayed anisotropic characteristics during compression, with axial compressive yield strength greater than radial. The core exhibited elastic behavior in the initial phase of compression, and the stress–strain changed nonlinearly, and then increased linearly. With the increase in the load and deformation, the stress gradually decreased and then slowly rose, indicating plastic deformation in the core. When the stress reached the tissue’s failure stress, it gradually decreased, as shown in Figure 5b. The stress–strain curves of the flesh indicate that, during compression, the axial and radial stress–strain trends of the flesh are similar. In the initial stage of compression, the stress–strain increases linearly, where the flesh exhibits elastic behavior. When the stress reaches its compressive strength, the flesh yields and compresses, at which point the flesh tissue is damaged, and the stress subsequently decreases, as shown in Figure 5c,d. After the experiment, the experimental data were processed by Origin 2021 (Origin Lab, Northampton, MA, USA) software, and the mechanical parameters of kiwifruit skin, pulp, and core were obtained, as shown in Table 1, which included Young’s modulus (stress–strain curve slope Tan α in elastic interval), tangential modulus (stress–strain curve slope Tan β in plastic interval), and biological yield stress (stress value at biological yield point).

### 3.2. Fruit Compression Damage

**Figure 6:**
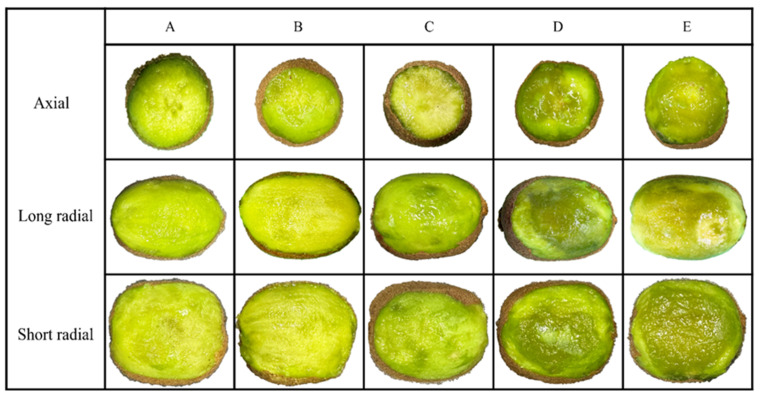
Browning of kiwifruit under different strain levels. (**A**) Control; (**B**) the strain level is 2.5%; (**C**) the strain level is 5%; (**D**) the strain level is 10%; and (**E**) the strain level is 20%.

The mechanical damage of kiwifruit is delayed, and the damage will not appear immediately after being squeezed [30]. According to the study by Mengjie Gao et al. [31] on the impact of compression damage on the optical properties of kiwifruit flesh, the CIELAB color values of kiwifruit flesh reach their maximum 16 days after compression. At this time, the bruising of the flesh is fully developed, and the color change becomes more pronounced. Therefore, after the experiment, the fruits were stored in a constant temperature and humidity box (temperature 24 °C, humidity 63%) for 16 days. Unprocessed fruits served as the control group, and the skin of the compressed area was removed to observe browning. The browning area under different strain levels is shown in Figure 6. With the increase in strain level, the area of fruit damage gradually expands. Under strain levels of 2.5% and 5%, no significant browning occurred in the axial, long radial, and short radial compression areas of the fruit. When the strain level reached 10%, the cells of kiwifruit flesh were damaged, leading to noticeable browning in the compressed area. At a strain level of 20%, the fruit began to exhibit plastic deformation due to compression, and the tissue browning area in the compressed region increased rapidly.

### 3.3. Finite Element Simulation and Experimental Results Analysis

**Figure 7:**
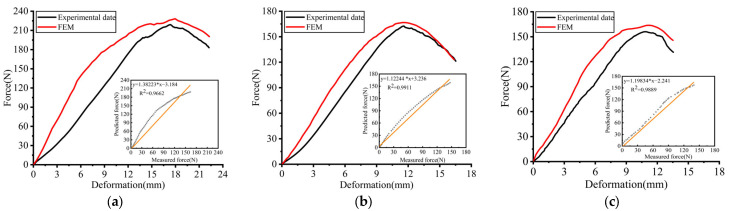
Comparison of the FEM results with the experimental results. (**a**) Axial; (**b**) long radial; (**c**) short radial.

To validate the effectiveness of the finite element model, the force–displacement curve obtained from the finite element simulation was compared and analyzed with the curve derived from the whole kiwifruit compression test. The force–displacement curves from both the finite element simulation and the experimental records are shown in Figure 7. It is evident from the figure that the mechanical properties of the fruit vary in different compression directions. This further indicates that the structural differences and material anisotropy of kiwifruit in various directions lead to differences in mechanical properties. In the initial phase of compression, the slopes of the force–displacement curves from both the simulation and experiment are highly similar, indicating that the finite element model captures the compressive elastic characteristics of kiwifruit well. As the compression displacement increases, slight deviations between the finite element and experimental curves, especially near the peak force, are observed. This discrepancy may be due to the finite element model not accounting for the microstructural characteristics of the fruit’s interior, such as seeds, tissue fluid, and seed chambers, as well as measurement errors in the experiment. The axial ultimate compression force is greater than the radial, with little difference in ultimate compression force between the long and short radial. This is consistent with previous studies indicating that the compression mechanical properties of fruits are closely related to the direction of compression [32,33]. To accurately describe the trend of compressive force during the fruit compression process, the predicted force in the compression yield stage is fitted with the experimental force. The results showed a high correlation coefficient for the finite element simulation compared to the compression experiment (axial 0.9662, long radial 0.9911, short radial 0.9889). The consistency between the predicted and measured force fitting curves indicated that the established finite element model can effectively simulate and predict the compressive mechanical behavior of kiwifruit.

**Figure 8:**
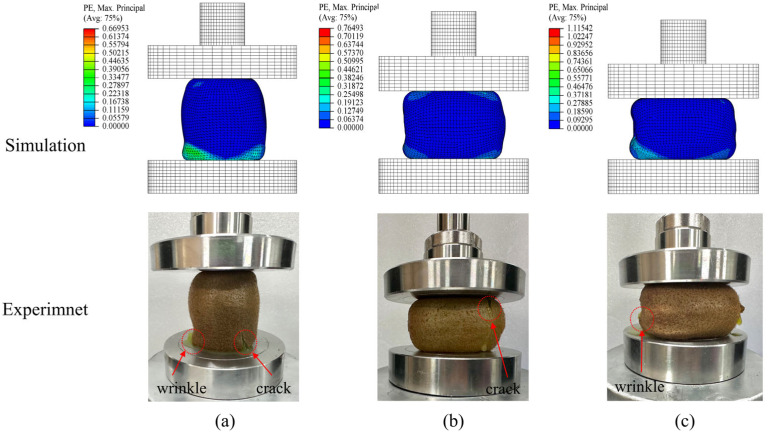
Compressive plastic strain. (**a**) Axial; (**b**) long radial; (**c**) short radial.

In order to intuitively analyze the deformation of fruit during compression, plastic strain (PE) was used to describe the amount of plastic deformation and the potential areas of rupture during the compression process. The plastic strain of the fruit during compression is shown in Figure 8. It can be seen that there is good consistency between the simulated and experimental results in the distribution area of plastic deformation. The fruit underwent plastic deformation to different degrees in the axial, long radial, and short radial directions and the deformation areas were mainly concentrated in the area in contact with the compression plates. With the increase in strain level, the kiwifruit is deformed to form wrinkles on the surface, the flesh cells are damaged, the local skin cracks, and a significant amount of tissue fluid leaks from the cracks. This indicates that the established finite element model can predict the deformation and rupture failure locations of the fruit to some extent.

### 3.4. Prediction of Internal Compression Damage Behavior in Fruits

#### 3.4.1. Analysis of Axial Stress Distribution Characteristics in Fruit

**Figure 9:**
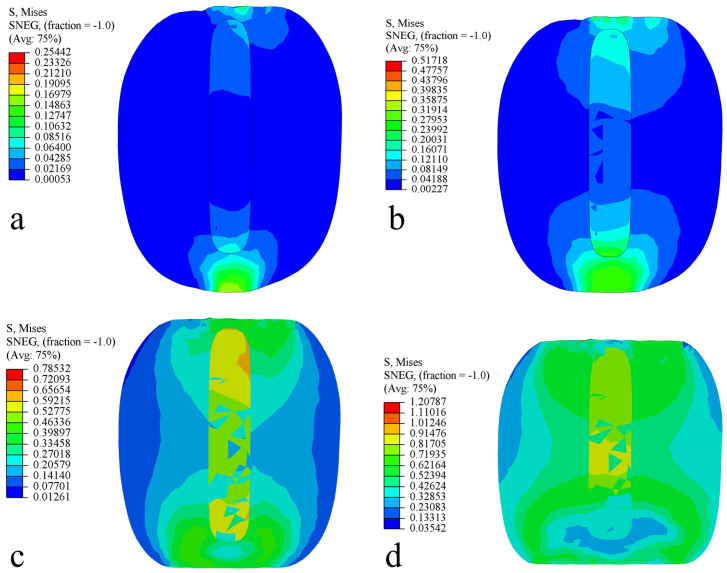
Von Mises stress distribution in the axial direction. (**a**) Strain level 2.5%; (**b**) strain level 5%; (**c**) strain level 10%; and (**d**) strain level 20%.

The axial distribution of Von Mises equivalent stress in the fruit under different strain levels is illustrated in Figure 9. During the compression process, stress is transmitted from the skin towards the flesh and the core. In the initial phase, stress spreads around the contact area with the compression plate and gradually decreases away from this region. The maximum equivalent stress is primarily concentrated in the skin area in contact with the compression plate. As the strain level increases, stress progressively shifts towards the core area, and the equivalent stress area of the skin, flesh, and core also expands. At strain levels of 2.5% (displacement 1.65 mm, force 36.74 N) and 5% (displacement 3.29 mm, force 75.82 N), the maximum equivalent stress in the flesh area is less than the biological yield stress of the axial tissue of the flesh, 0.491 MPa, and there is no significant damage to the flesh. When the strain level reaches 10% (displacement 6.58 mm, force 147.81 N) and 20% (displacement 13.16 mm, force 213.31 N), the maximum equivalent stress in parts of the flesh in the contact area with the plate reaches its biological yield stress, and the fruit begins to yield to compression. The equivalent stress gradually transfers towards the core area and the internal stress within the core increases, resulting in plastic deformation. With the increase in compression displacement, the equivalent stress in the fruit increases, and the damaged area gradually expands, which is consistent with the results of the fruit compression test shown in Figure 6.

#### 3.4.2. Analysis of Stress Distribution Characteristics in the Long Radial Direction of Fruit

**Figure 10:**
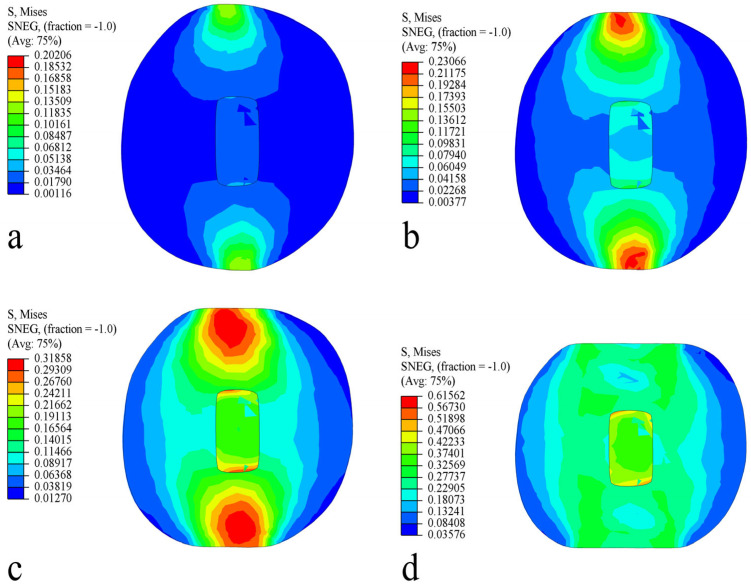
Von Mises stress distribution in the long radial direction. (**a**) Strain level 2.5%; (**b**) strain level 5%; (**c**) strain level 10%; and (**d**) strain level 20%.

Under different strain levels, the distribution of Von Mises equivalent stress in the long radial direction of the fruit is depicted in Figure 10. During the compression process, the stress on the flesh shows a symmetric distribution. As the compression displacement increases, the area of equivalent stress distribution in the flesh also expands. At strain levels of 2.5% (displacement 1.36 mm, force 21.51 N) and 5% (displacement 2.72 mm, force 47.512 N), the maximum stress in the flesh region is less than the radial biological yield stress of the flesh, 0.292 MPa, and there is no damage to the fruit. With increasing strain levels, the stress distribution gradually transfers towards the core area, and the area of stress distribution in the flesh tissue expands. When the strain level exceeds 10% (displacement 5.44 mm, force 101.97 N), the fruit gradually undergoes plastic deformation due to compression, and the damaged area also gradually expands. The damage trend of the fruit is consistent with the compression test results shown in Figure 6.

#### 3.4.3. Analysis of Stress Distribution Characteristics in the Short Radial Direction of Fruit

**Figure 11:**
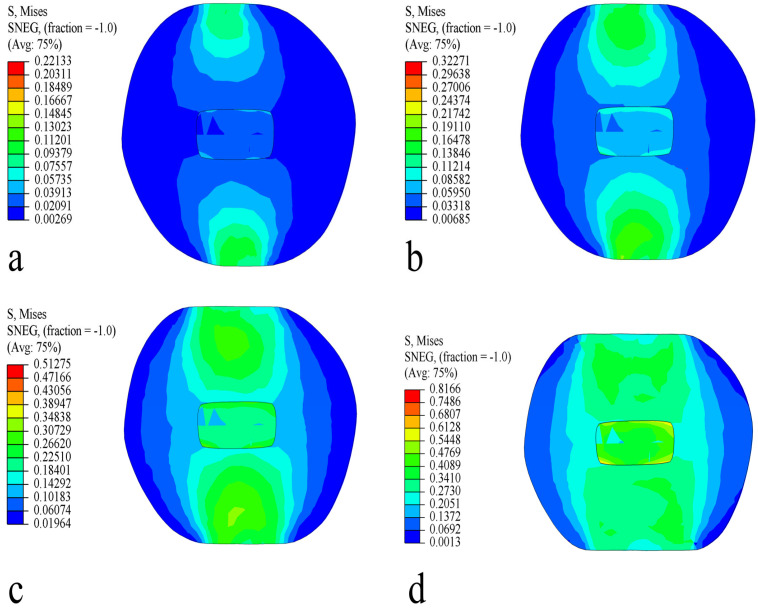
Von Mises stress distribution in the short radial direction. (**a**) Strain level 2.5%; (**b**) strain level 5%; (**c**) strain level 10%; and (**d**) strain level 20%.

Under different strain levels, the distribution of Von Mises equivalent stress in the short radial of the fruit is shown in Figure 11. The internal equivalent stress in the fruit is symmetrically distributed, gradually transferring from the skin to the core. With the increase in strain levels, the stress distribution gradually expands. At a strain level of 2.5% (displacement 1.27 mm, force 26.04 N), the internal stress distribution in the flesh is relatively uniform, with no obvious stress concentration. At a 5% strain level (displacement 2.55 mm, force 51.99 N), the stress in the area of contact with the compression plate gradually increases. When the strain level exceeds 10% displacement (5.12 mm, force 113.06 N), part of the flesh’s stress exceeds the biological yield stress of the flesh, leading to the fruit yielding to compression and gradually deforming.

The irregular shape of kiwifruit results in uneven stress distribution, and when subjected to external forces and local damage, it occurs easily in areas with large stress. During storage and transportation, fruits will be piled and squeezed. Therefore, it is necessary to analyze and study the force conditions of the fruit under different stacking methods to minimize the stacking compression damage to kiwifruit. Ashtiani et al. [29] have studied the compression damage of grapefruits and found that the number of grapefruits should not exceed 25 when placed vertically and not more than 31 when placed horizontally. Yancheng Lang et al. [24] analyzed the mechanical properties of prickly pear fruits under compression loads using the finite element method and found that the conditions for prickly pears to avoid mechanical damage is not to stack more than 125 fruits horizontally and not more than 1158 fruits vertically. Wei Liu et al. [21] stimulated and predicted the internal mechanical damage of pineapples under compression using the finite element method and found that when the pineapple was placed horizontally, there was no damage to the fruits when the number of stacks was less than eight.

Based on the above finite element simulation results, kiwifruit underwent no significant damage in the axial, long radial, and short radial directions when the strain level was less than 5%. When the strain level exceeded 5%, the flesh gradually yielded and deformed under compression, leading to damage. Thus, the compressive force at a 5% strain level can be considered as the damage threshold of the fruit. According to Newton’s second law, under ideal stacking conditions, the number of kiwifruits stacked should not exceed 77 in the axial direction, 48 in the long radial direction, and 53 in the short radial direction. Otherwise, the stress in the tissue of the fruits at the bottom could exceed the yield stress, leading to compression damage.

## 4. Conclusions

Compression damage is one of the most common damages to kiwifruit during harvesting, transportation, and storage, and its damage characteristics are difficult to observe directly with the naked eye. In this study, multi-scale modeling techniques were used to conduct physical and mechanical tests, and the geometric and mechanical parameters of each fruit component were obtained and a multi-scale geometric model of kiwifruit was established. Through the finite element method, the mechanical response behavior of kiwifruit under compressive loads was simulated. The simulation results provided an important basis for a deeper understanding of the damage mechanisms of kiwifruit under compressive loads. The main conclusions are as follows:

(1) Kiwifruit is anisotropic. The skin is brittle, while the flesh and core show elasto-plasticity. Compared with the core, the flesh has a lower compressive yield stress.
(2) The finite element simulation results are in good consistency with the experimental results. During compression, the stress distribution in the fruit is symmetric, with the maximum stress concentrated in the area of contact with the compression plate. With the increase in the strain level, stress gradually expands towards the core area. The destruction force of the fruit in the axial direction is greater than in the radial direction, with little difference in the compressive destruction force between the long and short radial directions.
(3) The flesh tissue of kiwifruit is more susceptible to damage during compression. When the strain level is less than 5%, no significant bruising occurs in the flesh or core in the axial, long radial, and short radial directions. The stress in the flesh and core tissues increases as the strain level increases. When the strain level exceeds 5%, some flesh tissues yield and are damaged.
(4) The finite element analysis method can effectively identify and predict the static compression load damage behavior of kiwifruit. However, the impact of kiwifruit’s maturity and variety on its mechanical properties still requires further study. Additionally, it is also the key direction for future research to establish scientific damage evaluation standards and obtain precise mechanical parameters of each fruit tissue. Future work will be devoted to the further optimization and refinement of the model to enhance the accuracy and applicability of damage prediction.

## Figures and Tables

**Table 1:** Properties of the kiwifruit.

Materials	Young’s Modulus (MPa)	Tangent Modulus (MPa)	Bio-Yield Stress (MPa)	Density(g/mm^3^)	Poisson’s Ratio
Skin	10.233	─	0.514	0.551	0.30
Core	4.499	1.381	1.306	1.154	0.30
Flesh Axial	2.305	0.967	0.491	1.248	0.40
Flesh Radial	1.346	0.642	0.292	1.248	0.40

## Data Availability

The original contributions presented in the study are included in the article, further inquiries can be directed to the corresponding author.
